# Acute high shear stress enhances fungal cell/substrate adhesion

**DOI:** 10.1128/spectrum.01952-25

**Published:** 2026-02-27

**Authors:** Md Adnan Karim, Dennis LaJeunesse

**Affiliations:** 1 Department of Nanoscience, Joint School of Nanoscience and Nanoengineering, University of North Carolina Greensboro 14616, Greensboro, North Carolina, USA; The University of Melbourne, Parkville, Victoria, USA

**Keywords:** hydrodynamic shear stress, *Candida albicans*, *Saccharomyces cerevisiae*, biofilm formation, Flo11p, ALS1p

## Abstract

Mechanical forces shape biological functions at all levels of life: biomolecules, cells, tissues, and organs. Shear flow, i.e., the transfer of momentum from regions of high momentum to those of lower momentum, influences the formation of microbial biofilms. Biofilms are complex structures composed of living cells and extracellular matrix that protects microbes from the host immune system, antimicrobial drugs, and physical damage. Despite the diversity of microbial morphology, behavior, and life cycle, biofilm formation is a conserved, multi-step process that follows a common sequence across microbial species. The initial step of this process is the adhesion of a microbe to a surface. This step is controlled by interfacial properties, including surface architecture, hydrophobicity, and composition, as well as environmental factors such as pH, nutrient availability, and physical/mechanical forces. Cell-surface adhesion molecules play a central role in the initiation of biofilm formation. In cellular fungi such as *Saccharomyces cerevisiae* and *Candida albicans*, adhesins such as Flo11p and ALS1p mediate attachment to host tissues and abiotic surfaces. The expression of these adhesins is tightly regulated by environmental conditions allowing fungi to rapidly adapt and colonize new niches. Once expressed, external shear forces modulate their activity, changing their conformation and organization in the cell wall and enhancing their adhesive properties. Previous work has demonstrated that shear forces below 2 Pa are needed for this mechanical enhancement to adhesion and that the force-dependent remodeling of the cell wall is a slow process. In this article, we demonstrate that exposure to acute high hydrodynamic shear stresses enhances and activates the adhesion of the two yeast species, *S*. *cerevisiae* and *C*. *albicans*. Using microfluidics and a new flocculation-based adhesion assay—termed as the bead adhesion precipitation assay—we show that a short exposure to a high threshold level of shear strain significantly enhances the adhesive capacity of yeasts.

**IMPORTANCE:**

Mechanical forces play integral roles in the fungal life cycle, impacting their metabolism, morphology, and biofilm organization. Sheer force has been demonstrated to control fungal cell adhesion by activating cell wall adhesion proteins, altering the tertiary structure of adhesion proteins, and reorganizing the display of these activated molecules on the surface of the cell. However, there is a limit. High levels of sheer force have been demonstrated to overcome cell-surface adhesion, resulting in a reduction of the number of cells on a surface. In this article, we show that cells exposed to high levels of shear rapidly respond to these forces and adapt by becoming more adhesive to surfaces. These results demonstrate that fungal cells adapt quickly to high shear and that this information needs to be considered when controlling biofilm formation and in the design of biomedical devices.

## INTRODUCTION

Mechanical forces are fundamental components of all living systems and are responsible for shaping biological functions at all levels of life: biomolecules, cells, tissues, and organs (1–3). Shear flow is the transfer of momentum from regions of high momentum to those of lower momentum. Fluids such as blood, interstitial fluids, and cytoplasm, as well as biological components within these fluids including biomolecules and cells, resist this transfer of momentum and therefore are subjected to shear forces (3). Even relatively low shear stress influences the transcriptional and morphological state of epithelial cells (2, 4–6). Microbial biofilms provide a physical barrier that protects microbes from the host immune system, antimicrobial drugs, and mechanical forces, thus enabling the persistence of infections and complicating treatment strategies (7–10). Despite the diversity of microbial morphology, behavior, and life cycle, biofilm formation is a conserved, multi-step process that follows a common sequence across microbial species (7, 10, 11). The initial adhesion of microbes is influenced by interfacial properties, including surface architecture, hydrophobicity, and composition, as well as environmental factors such as pH, nutrient availability, and physical/mechanical forces (12–17). In all cases, biofilm formation begins with the adhesion of a microbial cell to surfaces, which is mediated by adhesion proteins (10, 16, 18, 19). Adhesion molecules interact with surfaces through several different mechanisms, including ligand–receptor binding, hydrophobic interactions, and/or amyloid-like aggregation (1, 20–22). Fungal adhesion molecules, such as the ALS proteins in *Candida albicans* and Flo11p in *Saccharomyces cerevisiae*, are surface-expressed glycoproteins that mediate attachment to host tissues and abiotic surfaces (22–29). Their expression is tightly regulated by environmental conditions allowing fungi to rapidly adapt and colonize new niches. In *S. cerevisiae*, nitrogen starvation induces the expression of Flo11p, a key adhesion protein that enables cells to attach to abiotic surfaces (23, 25). Similarly, the expression of ALS1p in *C. albicans* is upregulated in response to environmental stress, including mechanical stresses such as those found on nanostructured surfaces or sublethal doses of antifungal drugs enabling biofilm formation and potentially enhancing virulence (30–33). Furthermore, these environmental conditions often control the quality and condition of the mature biofilm. These environmental influences often have long-term impacts on biofilm structure formation and stability; for instance, yeast biofilm formed under shear stress conditions is more stable and resistant to damage than those which are not (34, 35). In this article, we demonstrate that exposure to acute high hydrodynamic shear stresses enhances and activates the adhesion of the two yeast species, *S. cerevisiae* and *C. albicans*. Using microfluidics and a new flocculation-based adhesion assay—termed as the bead adhesion precipitation (BAP) assay—we show that a short exposure to a high threshold level of shear strain significantly enhances the adhesive capacity of yeasts.

## MATERIALS AND METHODS

### Microfluidic device fabrication

To make the microfluidic devices used in this work, we used a combination of 3D printing and soft lithography. We designed the device using the free and open-source OpenSCAD software (https://openscad.org/). Our design was converted to an STL file for its recognition in Chitubox, a free basic version of proprietary 3D slicer software (https://www.chitubox.com), which converted the STL file to a CTB file for specific resin-based 3D printing using an ELEGOO MARS 3 Resin 3D Printer (https://us.elegoo.com). The 3D-printed mold was cleaned in an isopropanol bath, post-printing treated with an ultraviolet lamp for 1.5 h, and dried overnight in an 80°C oven. Devices were cleaned, post-UV treated, and dried to remove the unreacted polymer from the mold surface (36). Failure to remove unreacted polymer from the mold surface properly inhibits the PDMS surface polymerization (36). The 3D molds were then used to make the PDMS device (SYLGARD 184 Silicone Elastomer Kit, Electron Microscopy Sciences) (37). Degassed PDMS mixture is poured over the 3D-printed mold and heat-treated in the oven at 80°C for 2 h; after 2 h, the mold and solidified PDMS are removed from the furnace, and the PDMS is gently peeled from the mold. The PDMS and glass slide were sonicated in a water bath for 15 min to remove dust from their surface; before use, the glass and PDMS were oxygen plasma-treated for 1 min to increase their surface energy (PE-100-RIE Plasma Etch System). Finally, glass and PDMS are bonded by applying manual pressure with the hand. The bond is due to siloxane bond formation between the glass and PDMS surface (37).

### Yeast used strains/culture

All strains (Table 1) were cultured in yeast extract peptone dextrose (YPD) medium at 37°C under 200 rpm agitation. Unless otherwise noted, all yeast strains were grown to mid-log phase with an OD_600_ between 0.4 and 0.6. We used a wild-type strain of *C. albicans* (ATCC 90028). We used three strains of *S. cerevisiae* (TBR5, TBR1, and TBR4) that expressed different levels of the Flo11p adhesion protein (17). For confocal microscopy and the flow cytometry experiments, we used the *S. cerevisiae* yeast strain L6906, which carried a hemagglutinin (HA)-tagged version of Flo11p. All *S. cerevisiae* strains were gifts from Todd B. Reynolds at the University of Tennessee.

**TABLE 1:** Yeast strains used in the experiments

Strain	Species	Phenotype	Genotype	Ref
TBR1	*S. cerevisiae*	Wild-type *FlO11*, normal adhesions	MAT_ ura3–52 leu2::hisG his3::hisG	(17)
TBR5	*S. cerevisiae*	*FLO11* knockout, reduced adhesion	*MAT*_ *ura3–52 leu2*::*hisG his3*::*hisG flo11*::*kanMX6*	(17)
TBR4	*S. cerevisiae*	Upregulated *FLO11*, hyperadhesive	MAT2 sf11:1 Kan Mx6ura 30 his 30 leu 20	(17)
L6906	*S. cerevisiae*	Regular levels of *FLO11*, HA epitope-tagged Flo11p	*MAT****a****ura3–52 his3*::*hisG FLO11*::*HA*	
90028	*C. albicans*	Wild-type strain	*Genotype A*	(38)

### Shear stress and cell adhesion microfluidic assay

To characterize the influence of shear stress on *S. cerevisiae* cell adhesion using our microfluidic device, we flushed the microfluidic channels with 50% isopropanol/water, followed by flushing with sterile water and YPD growth media periodically. Then 10 mL of cell suspension with a mid-log phase OD_600_ below 0.4 was vortexed for 5 min to distribute the cells. We loaded 10 mL the cell suspension in a syringe pump (New Era Pump Systems, Model 300) and flowed through the channels at the flow of 1.2 mL/min to generate the range of shear stresses (Table 2). Three images were taken from three different positions (start, middle, and end) of each channel through an inverted phase contrast optical microscope (Nikon, model TMS). The number of cells adhered there was counted manually. All experiments were performed in triplicate, and statistical significance was assessed using a two-tailed *t*-test.

**TABLE 2:** Physical characteristics of the microfluidic device

Channel	Width (mm)	Height (mm)	Shear stress (Pa)	Dwell time (ms)
I	0.5	0.4	1.34	0.3
II	0.32	0.32	3.26	0.1536
III	0.4	0.2	6.68	0.12
IV	0.2	0.2	13.35	0.06
V	0.18	0.17	20.53	0.0459

### Calculating shear stress and designing the mold

Shear stress (*σ*_*w*_) is a function of the solution flow rate (*Q*), viscosity of solution (*µ*), which for these experiments was considered 1, and channel dimension width (*w*) and height (*h*) (39):


$$\sigma_{w}=(6Q\mu )/wh^{2}.$$

As solution viscosity and flow rate remain constant, channel dimension establishes the stress force that the cells experience while moving through the channels. The dimensions and corresponding shear stress are defined by the mold or the microfluidic device (Table 2).

### Calculating adhesion index from microfluidic assay

To compare the adhesive qualities of different fungi, we calculated an adhesion index by normalizing the number of adhered cells to the total number of cells. To calculate the total number of cells, we measured the OD_600_ prior to each experiment and used a standard curve in which we normalized the OD_600_ to the total number of cells per unit volume as determined by CFU analysis.

### Bead adhesion precipitation assay

Yeast cells were cultured to an OD_600_ between 0.3 and 0.4 in YPD medium at 30°C. To measure the impact of shear flow on yeast cell adhesion, we subjected the yeast to a shear stress by flowing them through a 0.5 mm silicone tubing at different flow rates: 1.25, 10.0, and 15.0 mL/min to produce the following shear stresses: 1.7, 13.58, and 20.37 Pa. The length of tubing was adjusted to compensate for different exposure times: for 1.7 Pa shear stress, the tubing was 50 cm long; for 13 Pa shear stress, the tubing was 450 cm; and for 20 Pa shear stress, the tubing was 500 cm long. Cells were collected in an Eppendorf tube, to which 10 μL of a 100 mg/mL stock solution containing micron scale silica beads was added, ranging from 17 to 25 μm in size (Silk Mica: Mica Powder Plain, Mica −77019; TKB Trading); these particles were washed in dH_2_O five times and allowed to settle before removing the supernatant. This allowed small colloidal particles which might interfere with absorbance to be removed. These particles were added to each Eppendorf tube; the cell–bead mixture was incubated in a nutator for 5 min to facilitate cell bead interaction. The cell–bead solution was then allowed to settle for 10 min, after which 800 mL of cell–bead solution was transferred to a cuvette, and the OD_600_ was measured using a spectrophotometer (NanoDrop 2000c, Thermo Scientific). The difference between the OD_600_ before and after values normalized by the initial OD_600_ value. This number represents the adhesion index in the bead adhesion assay: the higher the number, the greater the adhesion. As a negative control test of the BAP assay, we treated the silica beads with PEG, which has been demonstrated to block cell adhesion (38). We examined the PEG-treated beads using scanning electron microscopy with energy-dispersive X-ray analysis for elemental composition and found an enrichment of carbon and oxygen on the surface (Fig. S1). We then performed the BAP assay with these PEG-silica beads and found no binding of cells to the surfaces of the silica beads (Fig. S2A through C) and low adhesion indices for all strains *S. cerevisiae* used in these experiments, including the hyperadhesive TBR4 strain (Fig. S2D).

### Quantification of Flo11p and ALS1p expression

We used *S. cerevisiae* strain L6906 (carrying an HA-tagged Flo11p) and *C. albicans* to quantify surface and total expression of Flo11p and Als1p under shear exposure. For surface staining, living cells were incubated for 30 min with the appropriate primary antibody: to detect Flo11p in *S. cerevisiae* strain L6906, we used the mouse anti-HA rRb-IgG (Developmental Studies Hybridoma Bank, AB_3105929), and to detect *Als1p* in *C. albicans*, we used the mouse anti-Als1, a gift of Lois L. Hoyer, University of Illinois (40). After washing, cells were then fixed using a 4% formaldehyde solution in phosphate-buffered saline (PBS) for 30 min. Washing steps were repeated, and cells were incubated with a goat antimouse secondary antibody conjugated with FITC (Jackson Labs, 115-226-075) in a blocking solution. PBS (1×) with 1% bovine serum albumin (Sigma, A32940) and 0.1% Triton X100 (Sigma, 9036-19-5). Confocal images of these samples were collected using an Olympus Evident FV3000 Confocal Microscope, and densitometric analysis was performed with ImageJ software (41). We performed a flow cytometry assay using a CytoFlex Beckman Coulter flow cytometer. All experiments were performed in triplicate, and statistical significance was assessed using a two-tailed *t*-test.

## RESULTS

### Microfluidic assay measuring adhesion index as a function of shear stress

**Fig 1:**
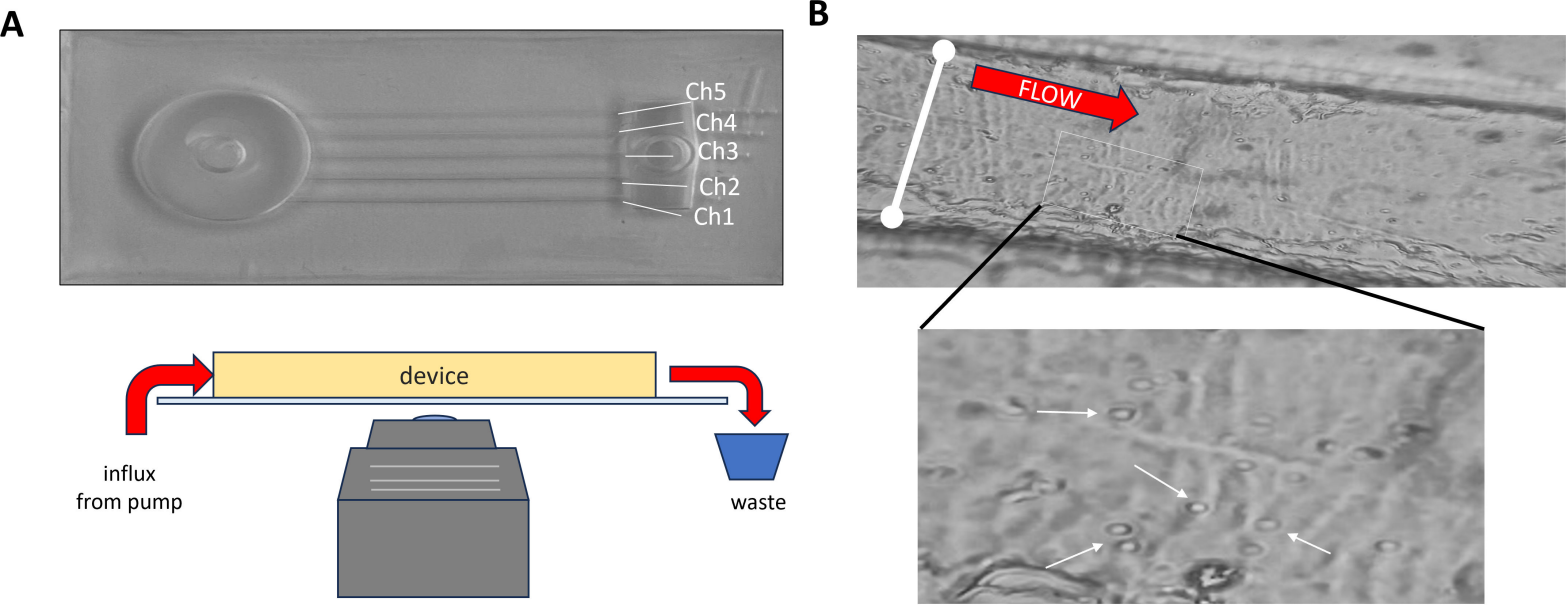
Microfluidic shear stress assay. (**A**) (Top) A micrograph of the device with a cylindrical reservoir to normalize the flow into the five channels. Shear stress (*σ*_*w*_) for each channel was calculated using the following formula *σ_w_ =* (6*Qµ)*/*wh*². In all the experiments, the same flow rate (*Q*) of 1.2 mL/min was generated by a syringe pump. *µ*, viscosity of solution ∼ water; h, height of the channel; *w*, width of the channel. The shear stress generated in each channel was calculated as Ch1 (the widest) = 1.34 Pa; Ch2 = 3.26 Pa; Ch3 = 6.68 Pa; Ch4 = 13.35 Pa; and Ch5 (the thinnest) = 20.53 Pa. (Bottom panel) The imaging setup. The PDMS shear stress microfluidic device was mounted on a large cover glass, and cells bound to the cover glass were then imaged with an inverted polarized microscope after flowing. (**B**) (Top panel) A representative image showing an example of the flow channel, in this case, wild-type *Saccharomyces cerevisiae* TBR1 cells in Ch4. The width of the channel is denoted by the white line and the direction of flow with the red arrow. The bottom panel is a detail of the rectangular panel in the top image. The white arrows denote yeast cells bound to the cover glass.

**Fig 2:**
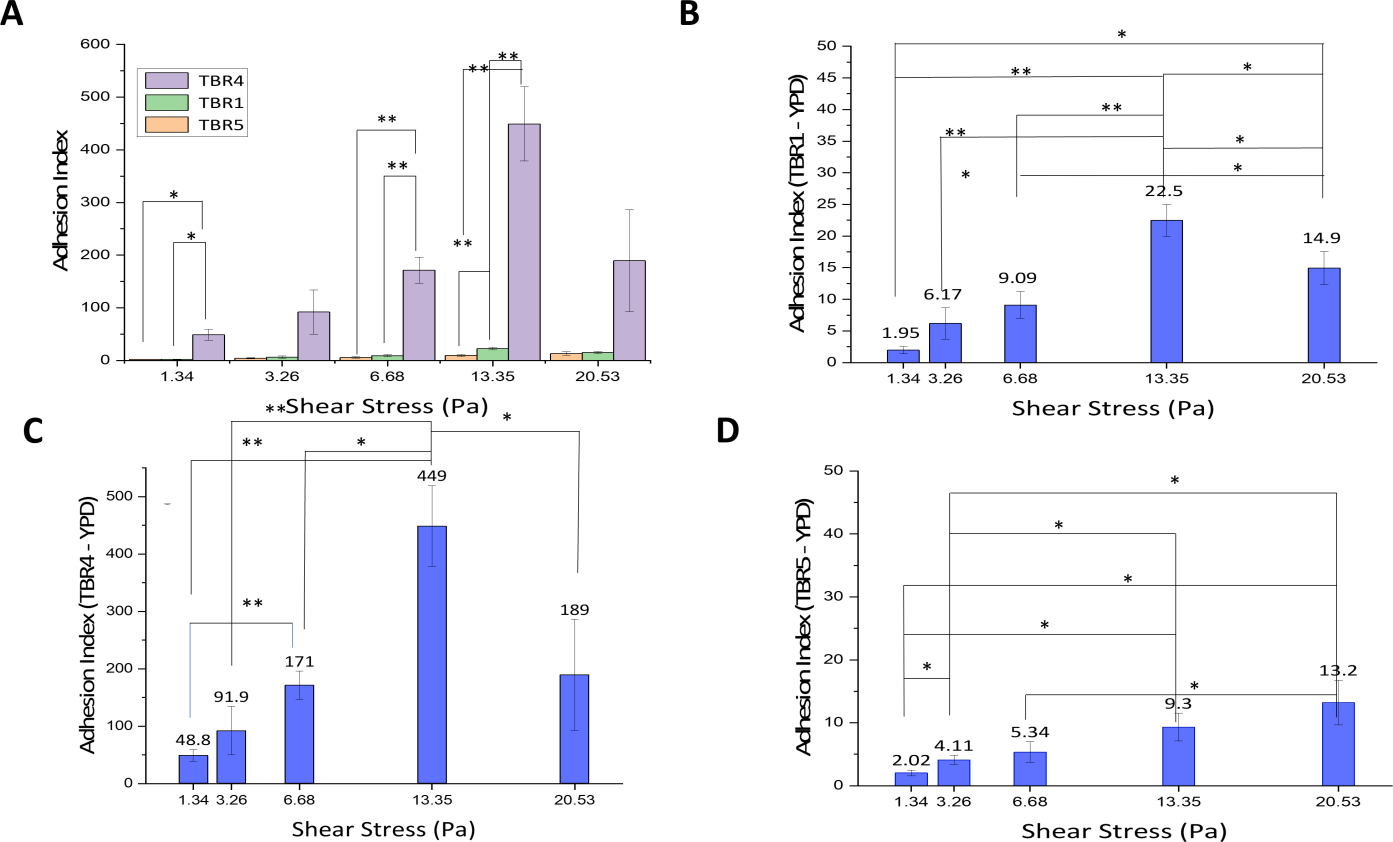
Stress-dependent adhesion of *S. cerevisiae* cells via a microfluidic channel-based assay. Adhesion index is defined as the ratio of the average number of cells adhering within each microfluidic channel to the total number of cells that flowed through that channel, multiplied by 10^6^. Statistical significance was assessed using a *t*-test and is indicated as follows: *P < 0.05, **P < 0.01, ***P < 0.001. (**A**) Summary of adhesion index among TBR5, TBR1, and TBR4 *S. cerevisiae* strains. (**B**) Wild-type *S. cerevisiae* TBR1 shows a rise in adhesion index with shear stress, reaching a maximum at 13.35 Pa; note the decline in adhesion at 20.53 Pa. (**C**) *S. cerevisiae* Flo11p-overexpressing strain TBR4 exhibits a higher adhesion index than TBR1 and displays a similar trend to TBR1 cells. (**D**) *S. cerevisiae* flo11p knockout TBR5 cells show incremental and significant changes in cell adhesion over the range of shear stress that do not follow the same trend as with adherent cells, suggesting a non-specific shear stress-driven interaction between cell and surface.

To determine how different environmental conditions impact adhesion, we grew *S. cerevisiae* cells to different culture conditions and then exposed them to different shear stresses by flowing through our microfluidic device (Fig. 1A). In these experiments, we flowed cells through channels of different widths in a PDMS microfluidic device and counted the number of cells adhered to the walls of this device (Fig. 1B). By using channels with different widths, we exposed the yeast to different shear stresses. We used the wild-type *S. cerevisiae* TBR1 strain, which expressed normal levels of the Flo11p, the primary adhesion molecule in *S. cerevisiae* (Fig. 2B) (16). For positive control, we used the hyperadherent, Flo11p overexpressing strain, *S. cerevisiae* TBR4, and for a negative control, we used the non-adherent Flo11p deletion strain, *S. cerevisiae* TBR5 (16). Adherent strains of *S. cerevisiae* (TBR1 and TBR4) exhibited a shear-dependent enhancement of adhesion when cells were exposed to shear stress greater than 13.35 Pa (Fig. 2A through C); both strains had significantly more adherent cells than yeast cells exposed to lower shear stress. In these same cell lines at higher shear flows (20 Pa), we observed a reduction in the number of cells, suggesting that this higher shear flow was enough to overcome flo11p cell-substrate adhesion (Fig. 2B and C). We observed similar trends in shear-dependent adhesion response (i.e., higher adhesion when subjected to shear stresses greater than 13.53 Pa when compared to lower shear stress conditions), when cells were cultured in a lower glucose medium (0.5% glucose versus 2%) and in a high-tonicity medium (YPD containing 0.1 M KCl), with adherent cells demonstrating the same pattern (Fig. S3 and S4). These results suggest that nutritional or solution tonicity is independent of this response. The hyperadherent TBR4 cells expressed an order of magnitude more cells than the wild-type TBR1 (compare scales between Fig. 2B and C) but exhibited a similar trend of higher number of adherent cells, which show a similar catch-bond/slip-bond pattern. The pattern of binding identified in these experiments mirrors the catch-bond/slip-bond patterns expressed by yeast previously identified in experiments using AFM single-cell experiments and examining yeast adhesion under lower shear conditions (1, 22, 34, 42, 43). The events described in this article are after an acute and rapid exposure (see Table 1), which suggests that the activation events occur almost immediately upon exposure to the mechanical stress. We also observe a slight non-threshold but significant increase in the number of cells adhering to the chamber walls as the shear stress is increased in the flo11p knockout strain, *S. cerevisiae* TBR5 (Fig. 2D). We do not observe a threshold response at the 13.35 Pa point, which suggests that there is a non-flo11p-dependent shear-dependent adhesion of the cell with the sides of our device, perhaps mediated by non-specific van der Waals interactions of cell wall components and the walls of the device.

### Bead adhesion precipitation assay

**Fig 3:**
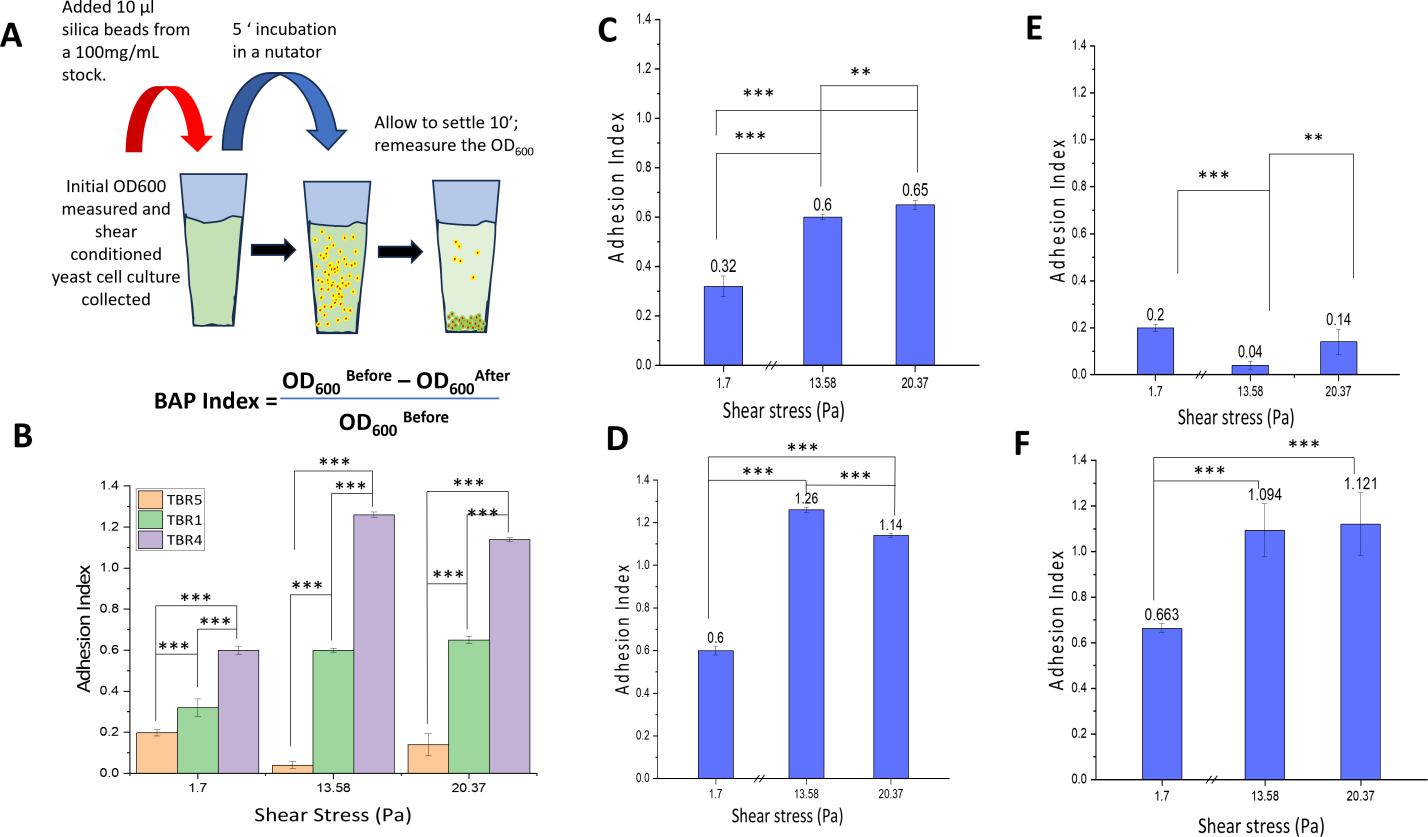
BAP assay adhesion assessment. (**A**) BAP assay schematic: the change in OD_600_ before and after bead exposure represents the BAP adhesion index. (**B**) Summary of BAP adhesion index among *S. cerevisiae* TBR5, TBR1, and TBR4 strains. (**C**) Wild-type *S. cerevisiae* TBR1 cells exhibit the shear-dependent BAP adhesion index, with higher adhesion index above 13.3 Pa shear exposure. (**D**) *S. cerevisiae* TBR4, overexpressing flo11p; TBR4 cells exhibit the highest BAP adhesion indexes with shear-dependent adhesion over 13.3 Pa shear exposure. (**E**) *S. cerevisiae TBR5* flo11p knockout cells exhibit uniformly low BAP indices with no shear-dependent patterns. (**F**) *C. albicans* cells exhibit a similar shear-dependent BAP adhesion exposure index. Statistical significance was assessed using a *t*-test: **P < 0.01, ***P < 0.001.

We also measured shear-dependent changes to yeast adhesion using a new adhesion assay called the BAP (Fig. 3A); this assay is similar in concept to flocculation adhesion assays and the MATH assay which measure relative surface hydrophobicity. Unlike the other assays, the BAP assay beads are not functionalized and therefore measure the binding of cells to an abiotic “standard” substrate (43–46). In these experiments, we used three different conditions: a low shear of 1.3 Pa, the threshold shear value of 13.35 Pa that was identified in the microfluidics experiment, and the 20 Pa shear. In the BAP assay, shear-conditioned yeast cells are incubated with large silica beads (Fig. S1) for 5 min on a rocker to facilitate cell–bead interactions, during which adhesive cells bind to the beads. The cell–bead mixture is then allowed to settle by gravity for 10 min, resulting in the formation of a cell–bead precipitate (Fig. 3A). We calculate an adhesion index by normalizing the difference in the optical density of the cell solution before and after exposure to the silica beads to the original starting OD_600_. The higher the index is, the greater the cellular adhesion. We observe that *S. cerevisiae* TBR1 cells and *S. cerevisiae* TBR4 cells exhibit an increase in adhesion after being exposed to shear forces greater than 13.6 Pa (Fig. 3B and C). In the negative control experiment, non-adherent *S. cerevisiae TBR5* cells with a deleted flo11p gene exhibit a low adhesion index (Fig. 3D). Wild-type *C. albicans* cells also exhibit a higher adhesion when exposed to shear stress above 13 Pa (Fig. 3F). These results mirror the results of our microfluidic experiments.

### Surface expression of fungal adhesion molecules

**Fig 4:**
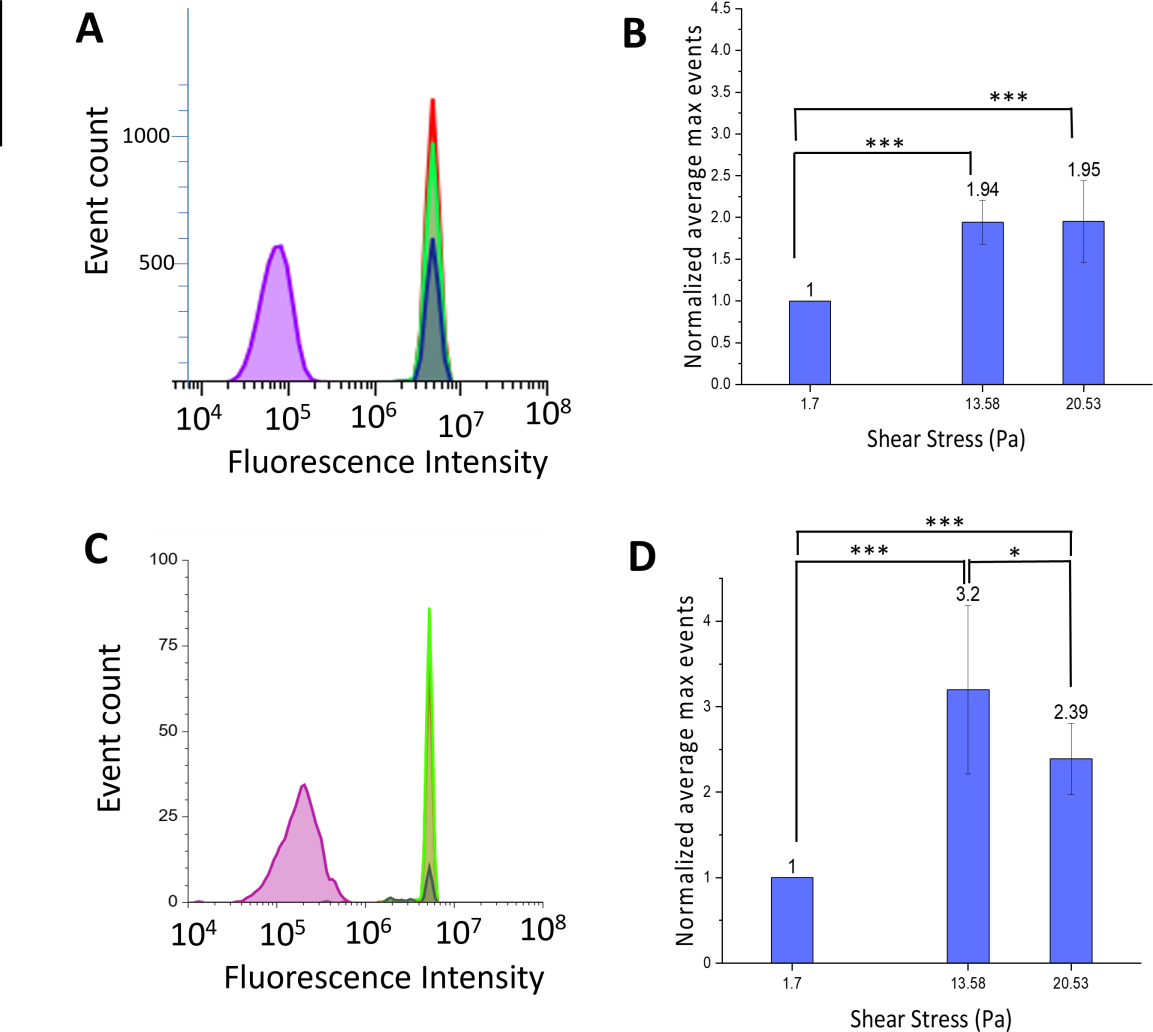
Shear-dependent expression of Flo11p and ALS1 adhesion proteins in *S. cerevisiae* and *C. albicans*. (A and C) Flow cytometry chromatographs: purple denotes untreated and unlabeled cells; dark green denotes cells treated with 1.7 Pa shear stress; chartreuse denotes cells treated with 13.58 Pa shear stress; and red represents cells treated with 20.53 Pa shear stress. (**A**) Flow cytometry chromatograph of levels of surface-expressed HA epitope-tagged flo11p adhesion protein in *S. cerevisiae* L6906 cells exposed to shear stresses. (**B**) A graph summarizing these experiments demonstrating significant differences between the number of cells expressing flo11p on their surface. (**C**) Flow cytometry chromatograph of levels of surface-expressed ALS1-labeled *C. albicans* cells exposed to shear stresses. (**D**) A graph summarizing the experiments demonstrating significant differences between the number of *C. albicans* cells expressing ALS1p on their surface. Statistical significance was assessed using a *t*-test and is indicated as follows: *P < 0.05, ***P < 0.001.

To determine whether the shear stress activated adhesion that we observed is due to changes and increase in the surface expression of adhesion molecules or due to an increase in the number of cells expressing the adhesion molecules on their surface, we labeled living cells with antibodies specific to these adhesion molecules to detect the expression of these molecules displayed on the cell surface (24, 25). For these experiments, we used the *S. cerevisiae* strain L6906, which expresses a Flo11p that has been tagged with the HA epitope tag, and we examined levels of ALS1 in *C. albican*s using a specific monoclonal antibody to this target (40). Using flow cytometry, we observed a twofold increase in the number of *S. cerevisiae* cells expressing Flo11p when subjected to shear stresses higher than 13 Pa (Fig. 4A and B). Densitometry of the confocal images of the *S. cerevisiae L6906* cells shows that there was little change in the level of expression among cells from the live cell experiment, with no significant difference among each condition (Fig. S5), which suggested that more cells expressed these surface moieties when subjected to shear stress greater than 13 Pa. To determine whether other cellular fungi also respond to the shear stresses, we examined the response of *C. albicans* to shear stress and examined the surface display of the ALS1p adhesion protein. ALS1p is a major adhesion molecule in *C. albicans* that is required for cell/substrate interaction and critical for biofilm formation (40, 47, 48). As with *S. cerevisiae* cells subjected to shear stress greater than 13 Pa, we observed a significant increase in the number of cells expressing Als1p, with almost three times as many cells expressing ALS1p on their surface when compared to lower shear stress (Fig. 4C and D). These results demonstrate a rapid increase in the number of cells expressing antibody-accessible surface targets in response to the shear flow. Fungal adhesins alter their conformation under mechanical stress, resulting in the formation of cross-β bonds. The formation of these cross-β bonds is essential for catch-bond formation as this conformation facilitates the formation of surface nanodomains containing clusters of the adhesin that have high affinity (1, 21, 35, 47). Furthermore, cross-β bond formation enhances the number of fungal cells within a biofilm two- to threefold, which mirrors the results we observe with expression of antibody-accessible surface targets. Our results suggest that acute exposure to high levels of shear force may trigger the formation of cross-β bonds and accelerate biofilm formation.

## DISCUSSION

Microbes live in a dynamic environment in which they experience many mechanical challenges, including forces imparted on the cells based on fluid flow, i.e., shear stress (1, 3, 18). Using a microfluidic adhesion assay and a new adhesion BAP assay, we found that yeast cells have a rapid (seconds/minutes) response to higher shear stress than previous studies (~13 Pa). Yeast cells exposed to laminar flow conditions a little over 1 Pa for prolonged periods of time (+2 h) increase their cell-substrate adhesion (1, 22, 34). Furthermore, yeast biofilms produced under these conditions are more resistant to mechanical damage, demonstrating a long-term effect on their adhesive properties. In these studies, the biofilm made of cells that were chronically exposed to applied shear forces over a longer time was more resistant to disruption. Single-molecule atomic force microscopy analysis of the *C. albicans* adhesin, Als5p, and other adhesins has demonstrated that a small amount of force is needed to unravel a portion of a fungal adhesion molecule and that once unraveled, these molecular tethers cluster to form adhesive nanodomains containing 10–100’s of high affinity cross-β bonds amyloid nanodomains, within cell wall rafts (21, 22, 29, 31, 34, 35, 43, 47). In *S. cerevisiae*, tension-dependent strengthening of these flocculin-based catch bonds is in part due to the clustering of Flo11p nanodomains within the plasma membrane, which enhances adhesion. The formation of these nanodomains involves quaternary interaction between Flo11p and does not necessarily involve increasing the number of adhesion molecules. In the case of biofilm formation, shear forces and perhaps other mechanical forces are predicted to activate adhesion in this manner. At the cell level, adhesion is regulated by catch-slip behavior. Under mechanical force, catch bonds become stronger due to the repeated activation and recruitment of activated adhesion molecules, enhancing adhesion, until a threshold force is reached, beyond which slip bonds weaken the interaction and promote detachment to relocate to more favorable environments (1, 21, 22, 43). The force-dependent enhancement of adhesion is slow due to the size/length of the molecules involved and the rate of diffusion of the activated adhesins across the cell wall (35).

So, why do we see a slightly faster response? This might be partially explained by our experimental methods. In our microfluidic assay, although the dwell time in the chamber is in the order of milliseconds (Table 1), the cells in this study had experienced shear stress for at least 5–6 min prior to being observed as we vortexed the cells prior to loading into the syringe to eliminate clumping; this treatment will generate sufficient shear force that increases the Flo11p flocculation rate almost instantly (22). The time in the 1 mm silicon tubing from syringe to microfluidic device is negligible, roughly 30–60 ms, making the total time (~5 min) only slightly shorter to the timescale (~20 to 30 min) observed previously for nanodomain formation and the demonstration of shear force enhancement of adhesion (1, 22, 34). Our data support previous work, and we do not believe that these differences between our work and previous work are significant (34, 43). We observe a clear catch-bond/slip-bond system in our microfluidic flow experiments (Fig. 2A).

We observe a higher threshold of force that enhances adhesion at ~13 Pa. Here the timing is interesting. In our microfluidic experiments, yeast cells encounter these higher-level shear forces and respond almost immediately (i.e., within milliseconds) both in our adhesion assays and in examining the surface expression of adhesion proteins. We observed a significant increase in adhesion after an acute exposure to 13 Pa of shear. Our results suggest a potential secondary activation mechanism, and this mechanism may involve a secondary and cooperative interaction between the surface and glycoproteins within the cell wall. In support of this is our observation of a slight but significant shear-dependent adhesion in the flo11p null *S. cerevisiae* TBR5 cells (Fig. 2C). The nature of the secondary mechanism is unclear, but perhaps it involves other components of the cell wall, such as the mannoproteins, the highly glycosylated stem of the adhesion molecules themselves, or some undescribed combination of these or other cell wall components. Such cooperative behavior in cell-substrate adhesion has been observed in some non-microbial mammalian systems (49, 50). The glycocalyx—the sugar/protein extracellular matrix found on the surface of many cells, including metastatic carcinomas such as glioblastoma multiforme—has been demonstrated to assist in integrin binding (49, 51). While integrin binding involves a ligand/receptor mechanism that is distinct from the amyloid-based mechanisms of fungal adhesins, both adhesive systems engage in the force-enhanced catch-bonding process (34, 52). Furthermore, the fungal cell walls are rich in glycoproteins, particularly mannoproteins, which may be acting to enhance cell-substrate adhesion (33).

Pathogenic fungi such as *C. albicans* are particularly adept at adhering to abiotic surfaces such as those found on biomedical devices, and in these situations, microbes derived from biofilm-contaminated biomedical devices result in systemic infections (1, 18). Within the human circulatory system, shear forces range between 0.1 and 9.5 Pa and mechanically demanding spaces such as the synovial joints of the knees; shear forces can reach higher up to 20 Pa under conditions of extreme mechanical loads and inflammation (3, 53–55). Microbial biofilms often form on the surfaces of biomedical devices, and in most cases, these biofilms form under dynamic conditions dominated by fluid flow (1). These adhesins control and drive both cell–cell and cell-substrate adhesions. Supporting these findings, recent studies exploring catheter design principles demonstrated that low shear stress areas with the device are associated with low biofilm formation potential, which is true for forming biofilm. However, our work demonstrates an unanticipated problem of shear activation of cells by high shear stress, which primes cells to colonize surfaces (56). Controlling high shear flow conditions also needs to be addressed in the design of biomedical devices and materials to control biofilm formation and lessen the potential of infection.

## Data Availability

The data supporting this study's findings are available within the article.

## SUPPLEMENTAL MATERIAL

Supplemental figures spectrum.01952-25-s0001.pdf Figures S1 to S5.

ASM does not own the copyrights to Supplemental Material that may be linked to, or accessed through, an article. The authors have granted ASM a non-exclusive, world-wide license to publish the Supplemental Material files. Please contact the corresponding author directly for reuse.
