# Supplementary material for: Acute high shear stress enhances fungal cell/substrate adhesion
Source: Microbiol Spectr. 2026 Feb 27;14(4):e01952-25. doi: 10.1128/spectrum.01952-25 (PMC13055298; doi:10.1128/spectrum.01952-25)
Supplement: Supplemental figures — Figures S1 to S5. [file spectrum.01952-25-s0001.pdf]

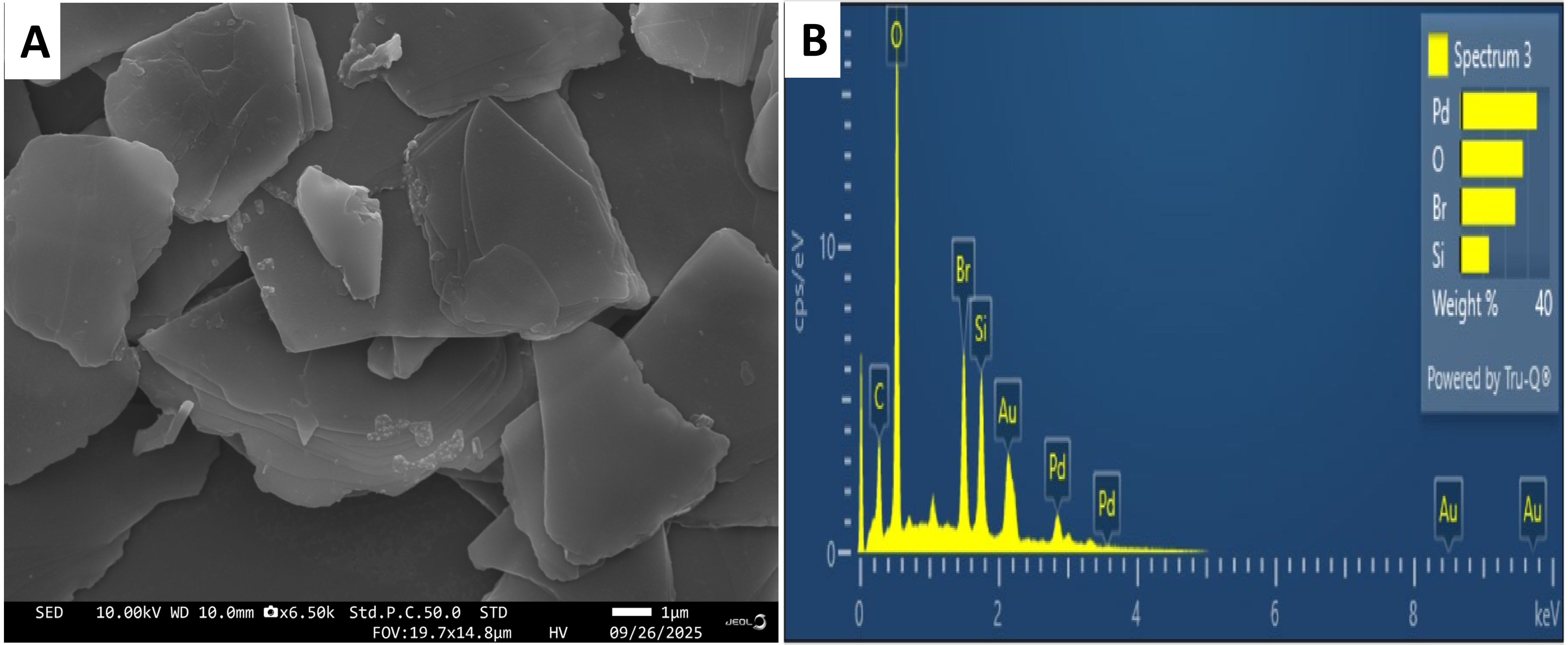

Supplementary Figure 1: Confirmation of PEG adsorption on silica beads for negative control experiments. (A) Scanning electron microscopy (SEM) images of silica beads after PEG adsorption. (B) Energy-dispersive X-ray spectroscopy (EDS) spectra showing carbon peaks, confirming PEG adsorption on the bead surface.

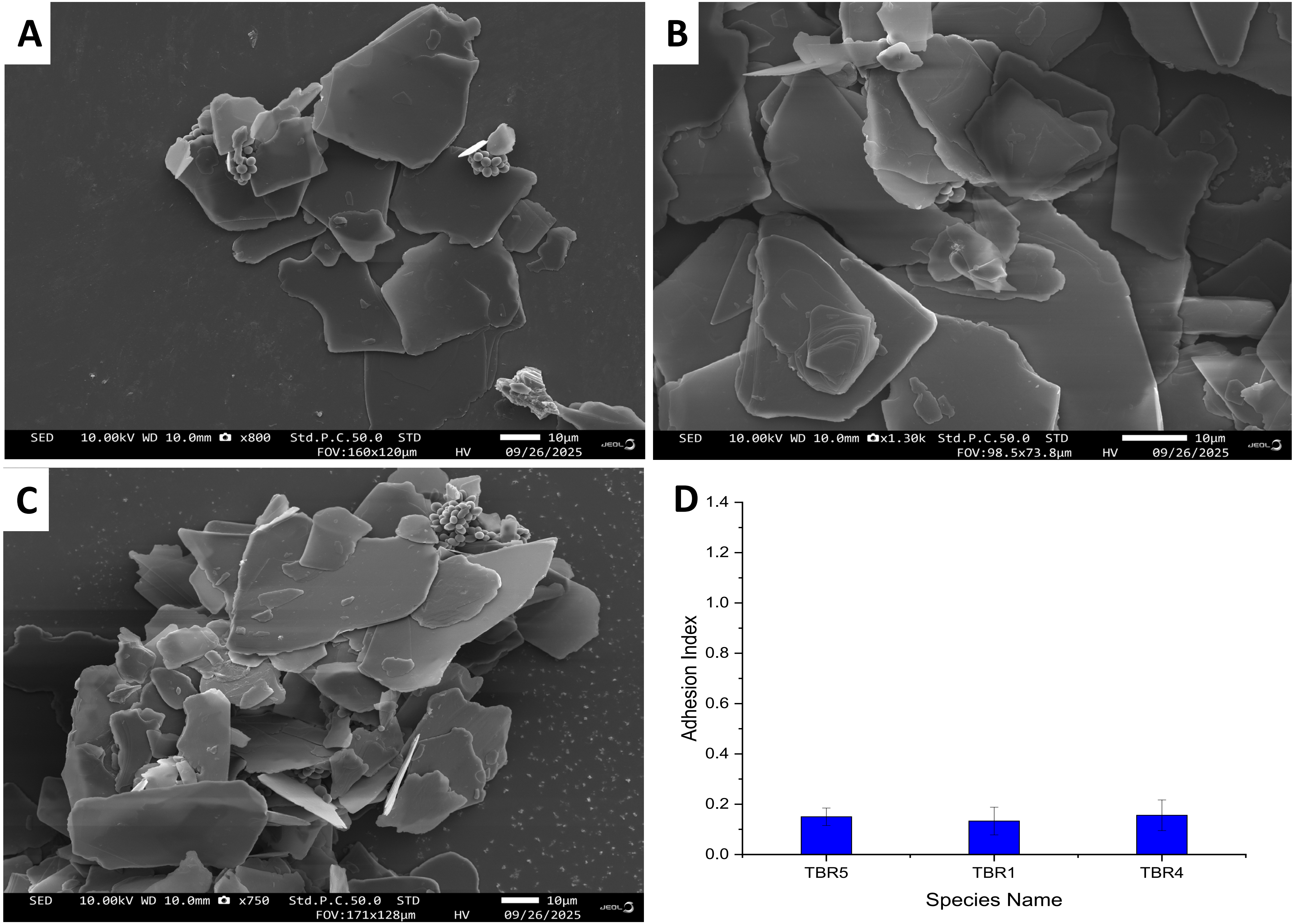

Supplementary Figure 2: PEG treated BAP assay beads – negative control experiment. A-C) SEM micrographs of silica beads used in the BAP assay. *A) S. cerevisiae* TBR5 *B)* Wild-type *S. cerevisiae* TBR1; *C) S. cerevisiae* TBR4 cells*;* D) BAP assay results for TBR5, TBR1 and TBR4 cells exhibit almost similar adhesion index with PEG adsorbed bead. With no observed significant difference.

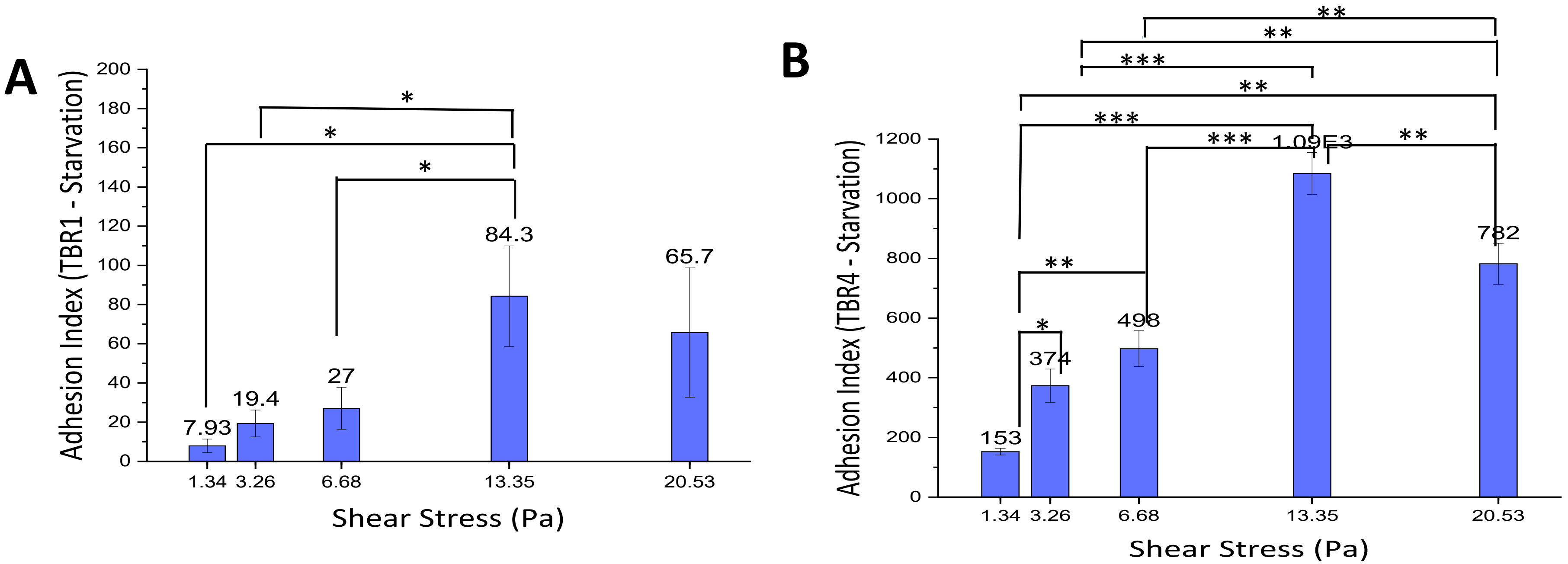

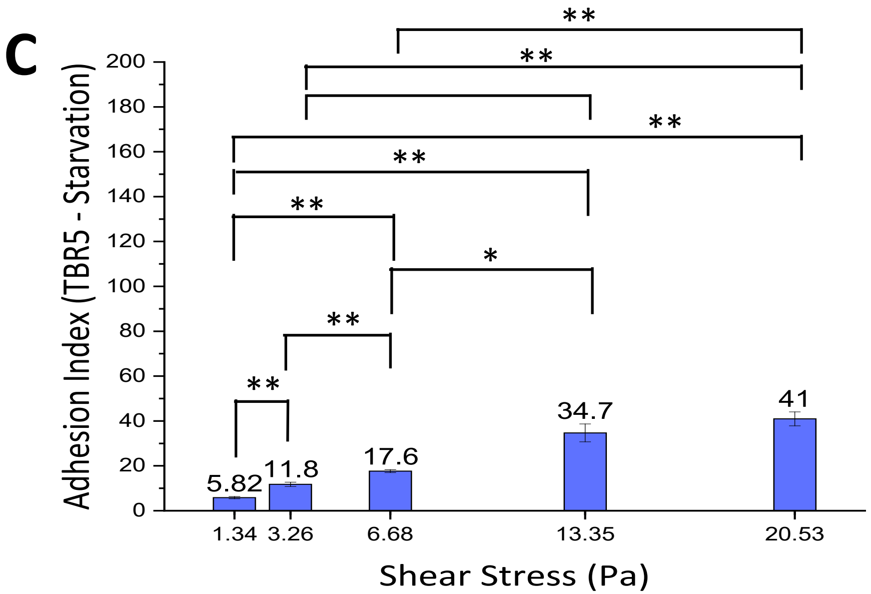

Supplemental Figure 3: Adhesion of *S. cerevisae* in shear stress microfluidic channel devise when cultured in low glucose (0.5%) YPD media. A) *S. cerevisiae* TBR1 cells; B) *S. cerevisiae* TBR4 cells; C) *S. cerevisiae* TBR5 cells. Statistical significance was assessed using a t-test and is indicated as follows: $p < 0.05$ (*), $p < 0.01$ (**), $p < 0.001$ (***).

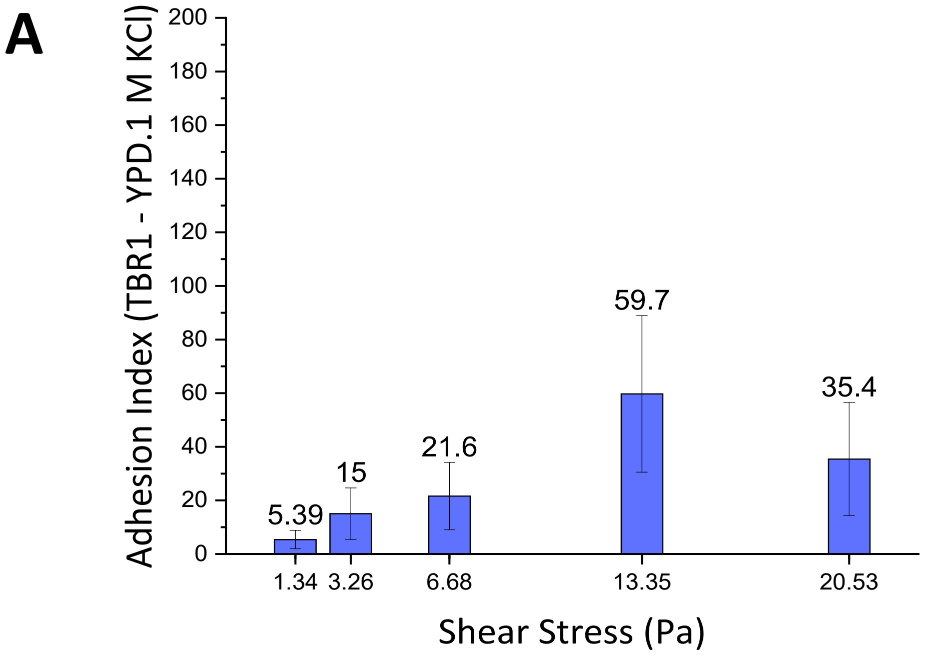

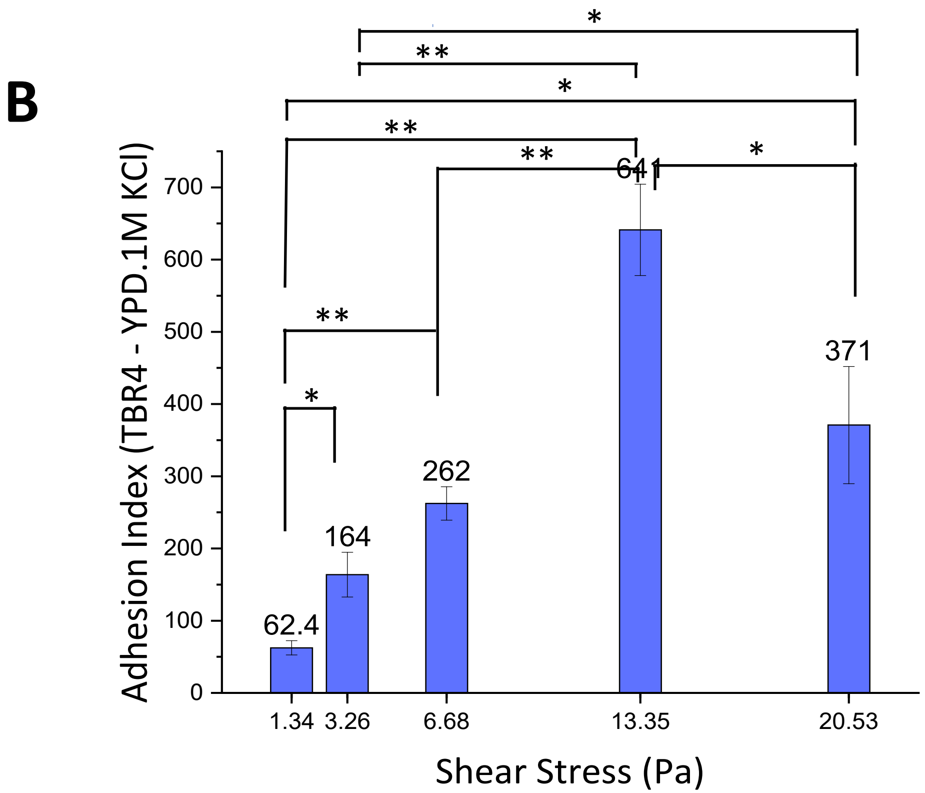

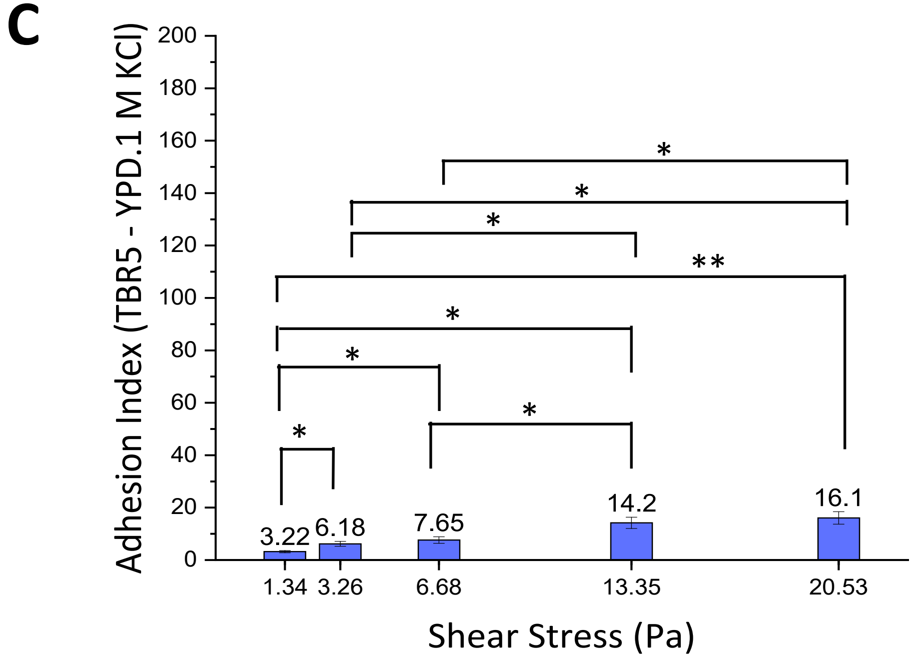

Supplemental Figure 4: Adhesion of *S. cerevisae* in shear stress microfluidic channel devise when cultured in a YPD medium containing 0.1M KCl. A) *S. cerevisiae* TBR1 cells; B) *S. cerevisiae* TBR4; C) *S. cerevisiae* TBR5 cells. Statistical significance was assessed using a t-test and is indicated as follows: $p < 0.05$ (*), $p < 0.01$ (**), $p < 0.001$ (***).

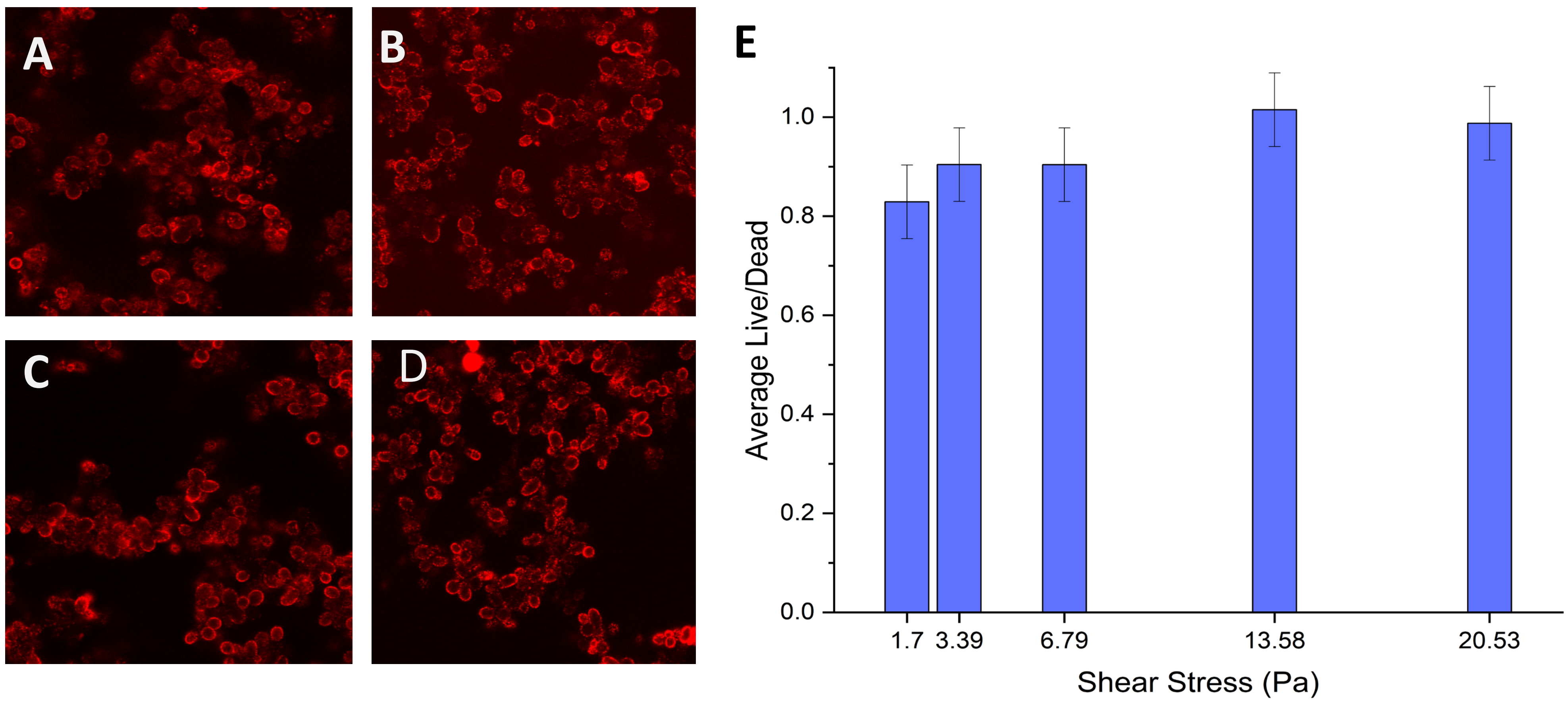

Supplementary Figure 5: Levels of Surface expressed HA-tagged flo11p per cell is unchanged regardless of shear. A) Live cell incubated with primary antibody after 13 Pa shear exposure. B) Live cell incubated with primary antibody after 20 Pa shear exposure. C) Fixed cell incubated with primary antibody after 13 Pa shear exposure. D) Fixed cell incubated with primary antibody after 20 Pa shear exposure. E) a graph summarizing the ratio of average densitometric value on confocal images, no significant difference in the levels of flourescence.
